# The judet quadricepsplasty for elderly traumatic knee extension contracture: a case report and review of the literature

**DOI:** 10.1051/bmdcn/2019090321

**Published:** 2019-08-27

**Authors:** Benjamin Tze Keong Ding, Suheal Ali Khan

**Affiliations:** 1 Department of Orthopaedic Surgery, Tan Tock Seng Hospital 11 Jalan Tan Tock Seng 308433; 2 Department of Orthopaedic Surgery, Khoo Teck Puat Hospital 90 Yishun Central Singapore 768828

**Keywords:** Judet Quadricepsplasty, Quadricepsplasty, Knee Contracture, Extension Contracture

## Abstract

Traumatic injuries to the knee are frequently complicated by extension contractures. The Judet Quadricepsplasty allows for controlled, sequential release of extrinsic and intrinsic knee contracture components while reducing the potential for iatrogenic quadriceps rupture. We document our institutions experience with this procedure and a systematic review of the current literature. We followed up on an elderly patient with posttraumatic flexion contracture that failed conservative management and underwent Judet Quadricepsplasty. Her knee range of motion improve dramatically from 20 degrees of flexion to 100 degrees of flexion. There was a residual extension lag of 5 degrees which did not impede on the patients daily activities. A review of the literature was performed and relevant data from 12 articles was extracted. The procedure was mainly performed in young adult males in most previous studies and the range of motion improvement ranged from 51° to 110°. Wound infections were the most common complication but otherwise other complications and severe extension lag were rare. The Judet Quadricepsplasty is a useful procedure for severe extension knee contractures that has failed conservative management in all age groups of patients. It is associated with significant increases in range of motion with low rates of complication or extension lag.

Diagnostic IV

## 1. Introduction

Knee extension contractures are a common complication of severe trauma to the knee, particularly in the supracondylar region. The extensive soft tissue and bony injury, dissection and manipulation during surgery and delayed rehabilitation all contribute to increased stiffness of the knee joint. Previous studies indicate that the normal individual requires less than 90 degrees of knee flexion for normal gait and slopes, 90 to 120 degrees of flexion for staircases, seating in chairs and approximately 135 degrees of flexion for a bath[1].

Thompson[2] and Judet[3] techniques of quadricepsplasty and their modifications have been described to correct recalcitrant cases of knee extension contractures. Thompson quadricepsplasty in adults has been shown to cause significant knee extension lag when quadriceps tendon lengthening is performed. Judet’s quadricepsplasty has been shown to have better outcomes and reduced occurrence of extensor lag as it allows for a controlled, sequential release of contracted components and a proximally based quadriceps muscle slide.

Our study showcases this technique’s utility for elderly traumatic extension contractures and performs a systematic review of the literature. The authors had obtained the patient’s informed written consent for print and electronic publication of the case report and institutional review board approval was obtained for the purpose of the study.

## 2. Case report

A 61-year-old female presented with Gustilo-Anderson Grade 3C compound fractures of the distal femur and Shatzker five tibial plateau fracture. She underwent initial debridement and a spanning external fixator in the acute setting and subsequently definitive fixation with a retrograde femoral intramedullary nail and lateral and posteromedial proximal tibia plates, with a bipedicled gastrocnemius flap and split skin grafts inset over the large skin defect in the same setting. The healing was complicated by tibia plateau fracture and she underwent a planned bone grafting 3 months later.

**Fig. 1:**
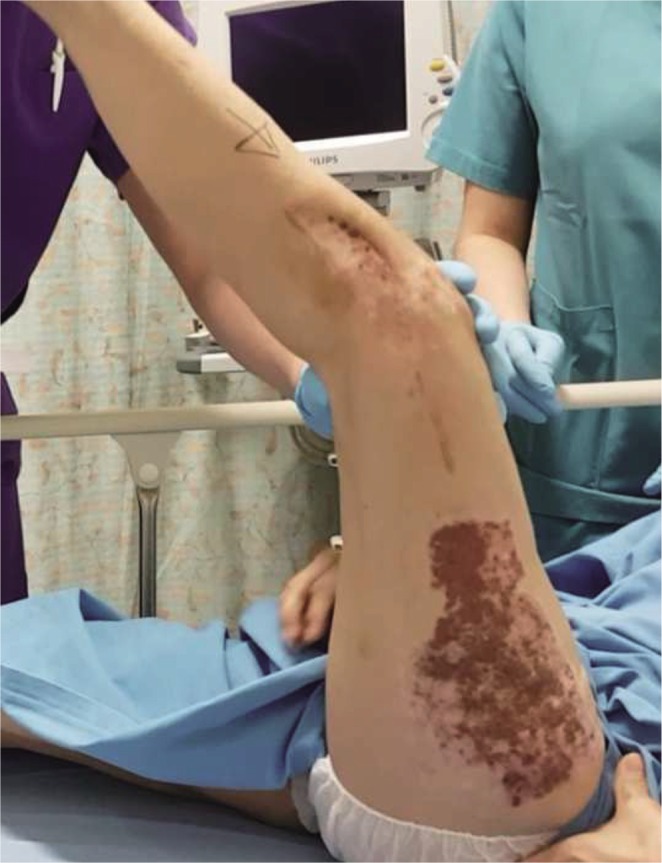
Pre-operative range of motion to 20 degrees.

Bone union was achieved at 5 months post operatively, but the knee range of motion was limited from 5 to 20 degrees as shown in Figure 1. Aggressive physiotherapy was commenced but failed to improve the range of motion. A Judet quadricepsplasty and removal of implants was subsequently performed 12 months after the initial surgery and 9 months after the secondary bone grafting surgery.

**Fig. 2:**
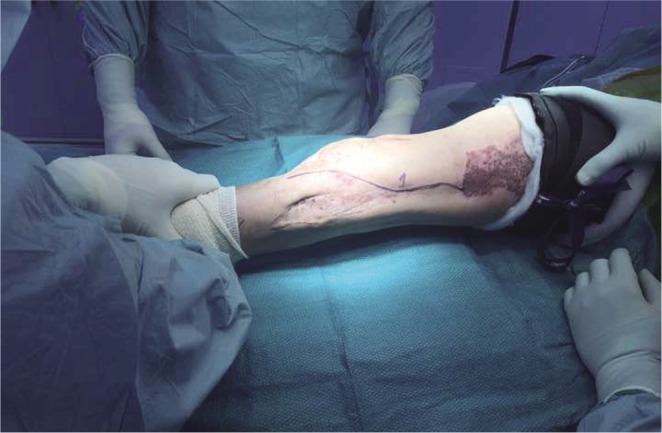
Lateral incision.

**Fig. 3:**
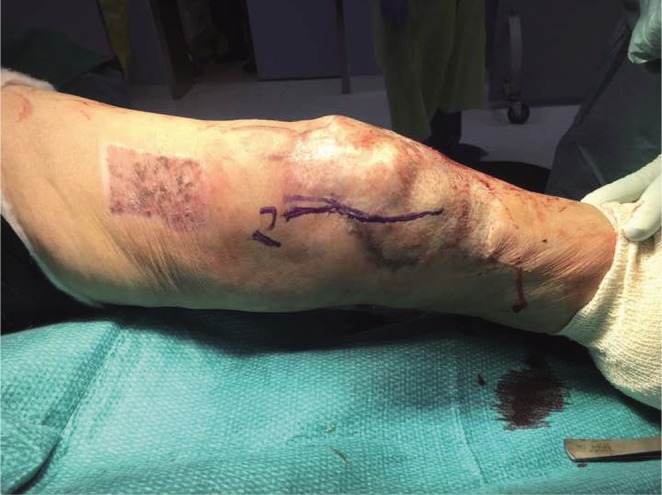
Medial S-shaped incision.

The anaesthetic team inserted an epidural catheter for continuous epidural anaesthesia prior to surgery and was retained in situ for the first week. Intravenous Cefazolin was given 1 hour prior to initiation of surgery and continued on for 24 hours. In supine position under general anaesthesia, the leg was cleaned and draped above the level of the anterior superior iliac spine (ASIS) to the foot. A sterile tourniquet is applied and the initial skin incisions are marked out; along the lateral intermuscular septum to the lateral aspect of the patellar tendon and a medial parapatellar S-shaped incision as depicted in Figures 2 and 3.

The lateral incision was made first directly through the skin, fascia and muscle preserving the inter-connective tissue between the fascia and the skin to preserve dermal blood supply. The knee joint was entered *via* a lateral parapatellar capsular incision and the intra-articular adhesions between the femoral condyles, tibia and patella were released.

**Fig. 4:**
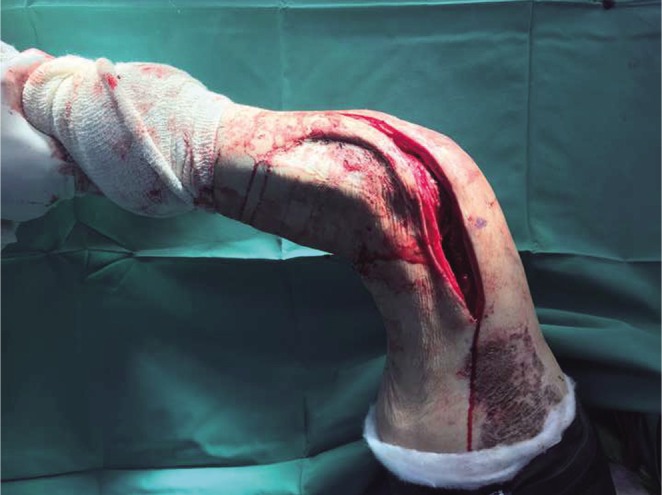
Flexion to 60 degrees after medial and lateral periarticular capsular releases.

A medial parapatellar approach was then utilized for adhesiolysis of the medial aspect of the knee. A simultaneous partial release of the medial collateral ligament (MCL) and medial capsule was performed. Under radiological guidance, the posteromedial tibial plate was removed to prevent possible impingement during flexion. At this point of time, the knee was ranged and flexion had improved to 60 degrees as shown in Figure 4.

The tourniquet was then removed and the lateral incision was then extended to the level of the Greater Trochanter. The rectus femoris origin was released at its origin *via* the same lateral incision, a modification of the original Judet’s procedure.

**Fig. 5:**
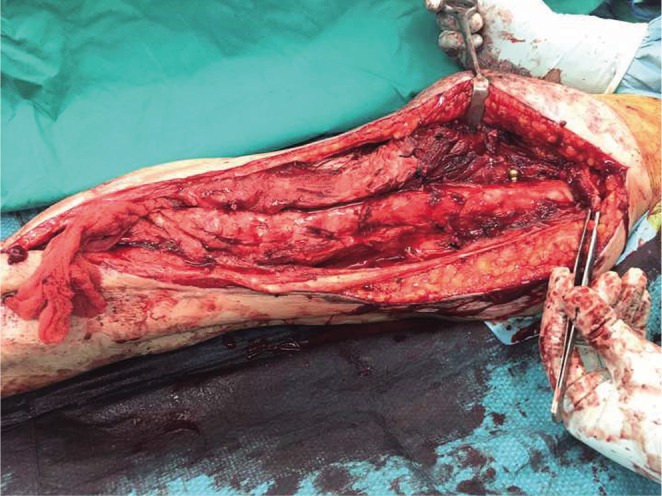
Quadriceps muscle slide.

The quadriceps muscle slide was then performed by elevating the quadriceps muscle off the femur along the intramuscular septum using an extra-periosteal approach as shown in Figure 5. Careful cauterization and ligation of the perforating vessels was performed at this juncture to prevent significant blood loss.

**Fig. 6:**
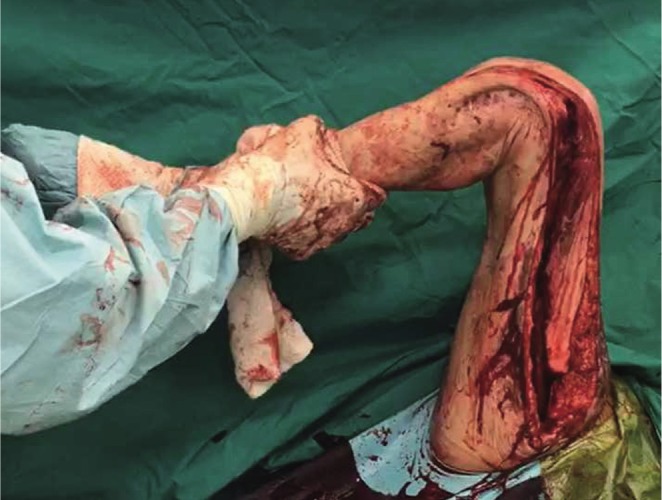
Knee range of motion increased to 120 degrees after Judets Quadricepsplasty.

Fractional lengthening of the fascia lata and anterior fascia of the thigh was then performed by making multiple transverse incisions at multiple levels. The intramedullary femoral nail was then removed. At this point, maximal knee flexion of 0 to 120 degrees was achieved as shown in Figure 6. The medial and lateral incision’s subcutaneous tissue and skin were then closed over vacuum drains with the capsule intentionally left open to prevent recurrent capsular adhesion formation.

An epidural catheter provided analgesic relief for 5 days post-surgery before gradually being weaned off and converted to oral analgesia.

Continuous Passive Motion (CPM) was initiated in the postoperative care unit and continued for 24 hours for the first week. Electric muscle stimulation of the quadriceps muscles, aggressive physical therapy and cycling exercises were started on the 3rd post-operative day and the patient was allowed to ambulate on the 6th day post-surgery, after the epidural catheter had been removed.

Her drains were removed on post op day 4 and 5 when the drainage amount was minimal. Regular monitoring of her haemoglobin levels showed a drop of 1.0 *g/dL* on the first post-operative day requiring blood transfusion. The haemoglobin levels were subsequently maintained.

**Fig. 7:**
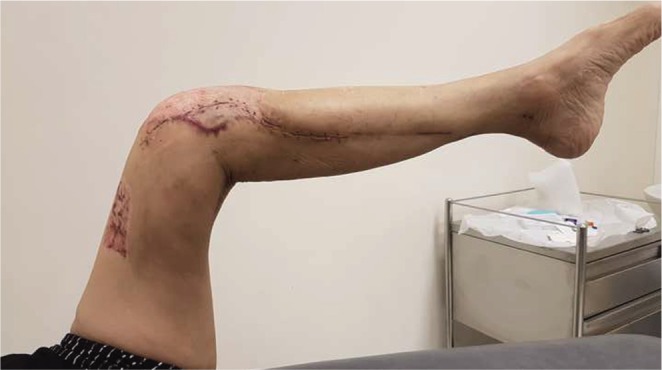
Knee range of motion decreased post operatively to 90 degrees at 3 months follow up.

Removal of sutures was performed at 3 weeks where the wounds had healed well and there was no evidence of dehiscence or skin edge necrosis. At 3 months follow up, the patient was able to ambulate and had knee range of motion from 0 to 90 degrees without extension lag as seen in Figure 7. This represented an increase in range of motion of 75 degrees and the patient was able to climb stairs and return to daily activities.

## 3. Discussion

We performed a literature search of three databases, PubMed, Scopus and Web of Science, using the keywords, “quadricepsplasty”, “Judet Quadricepsplasty” which were subsequently matched with search terms “knee contracture”, “extension contracture”. Published articles between January 1982 and January 2017 were retrieved and their titles, abstracts and full texts were reviewed for appropriateness.

Inclusion criteria included: (1) Randomized-controlled trials, longitudinal cohort and retrospective studies, (2) Assessment of surgical outcomes of Judet Quadricepsplasty in patients with knee contractures, (3) Full-text availability, (4) Manuscript written in English Language. Exclusion criteria included: (1) Study population with majority being under 21 years’ old, (2) Studies conducted outside the time frame between 1982 to 2017, (3) Meta-analyses and Systematic Reviews (4) Outcomes of other techniques of quadricepsplasty.

**Fig. 8:**
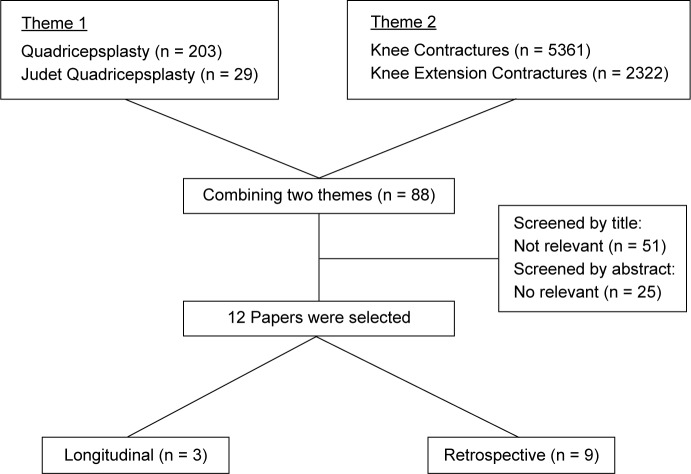
Flow diagram of literature review.

We scanned the reference first by titles and published abstracts, to exclude non-relevant articles. Of the original 88 articles that met the selection criteria, we subsequently reviewed the available full texts to identify 12 articles that were summarized in this paper. The search process is as documented in the flow chart in Figure 8.

The data from each article was compiled into a standardised table (Table 1), which included: Study information (Author(s), date of publication, study design, region, sample size), patient demographics (age, proportion of male gender), etiology of contracture, increase in range of motion (including both pre-operative and post-operative range of motion and remaining extension lag), surgical and post-operative complications, need for secondary manipulation under anaesthesia and duration of follow-up. Data that was not available or unspecified from the full texts were indicated with “n.a” (not available) in the tables.

**Table 1:** Table of systematic review.

S/N	Author	Study Design	Region	Sample Size	Demographics		ROM Improvement (Pre-op to post-op ROM)		Extension Lag	Cause of Contracture	Complications	Post Op Manipulation under Anaesthesia	Follow-up Duration
					Age (mean years)	Male Gender (%)							
1	Bari 2015	Retrospective review	Bangladesh	32	26	100	75° (15° to 90°)		None	12 – Ilizarov	6 – Wound infections	n.a	24 months
										20 – Internal fixation								

2	Mahran 2014	Longitudinal Study	Egypt	19	30	95	93.5° (22.6° to 116.1°)		n.a.	7 – Ilizarov	0	n.a.	6 months
										11 – External Fixator			
										2 – Internal Fixation			

3	Oliveira 2011	Retrospective review	Brazil	45	32	65	51° (34° to 85°)		15 × 1	6 – Ilizarov	5 – Surgical site in-fections	9 (14%)	24 months
										27 – Internal Fixation			
										12 – Casting			

4	Lee 2010	Longitudinal Study	Korea	10	39.5	80	69° (25° to 94°)		5 × 1	10 – Femoral fractures	2 – Pin track infections	n.a.	52 months
									10 × 1				

5	Masse 2008	Longitudinal Study	Italy	21	29.6	71	72° (23° to 95°)		n.a.	14 – Ilizarov	2 – Deep sepsis	n.a.	101 months
										3 – Internal Fixation	1 – Quadriceps tendon rupture		
										3 – External Fixator	1 – Skin necrosis		
										1 – Casting	1 – Lateral femoral condyle fracture		

6	Rose 2005	Retrospective review	Jamaica	4	44.8	25	90° (5° to 95°)		None	2 – Ilizarov	0 complications	None	8 months
										1 – Severe Osteoarthritis			
										1 – Tibia plateau fracture			

7	Ali 2003	Retrospective review	England	10	35.6	n.a.		55° (33° to 88°)	10 × 1	10 – External Fixator for dis-tal femoral fractures	1 – Haematoma	7 (70%)	24 months
											1 – Infection		

8	Belleman s 1996	Retrospective review	Belgium	16	30.4	44	68° (23° to 91°)		15° × 4	4 – Internal fixation of femoral fractures	1 – Deep infection	n.a.	22 months
										2 – External fixator of femoral fractures	1 – Compartment syndrome		
										1 – Internal fixation of patella	1 – Recurrence requiring arthrodesis		
										1 – Casting of femoral fracture			
										4 – Ilizarov limb lengthening			
										3 – Sequelae of IM injection			
										1 – Post knee replacement			

9	Ebraheim 1993	Retrospective review	U.S.A	12	43	73	53° (38° to 91°)		5° × 3	12 – Femoral Supracondylar fractures	1 – Requiring primary flap and immobilisation	n.a.	25 months
									10° × 1				
									15° × 1				
									30° × 1				

10	Merchan 1992	Retrospective review	Spain	21	31.8	86	57° (27° to 84°)		10° × 6	21 –Fractures	1 – Wound infection	3 (14%)	68 months
									15° × 1	16 – Internal fixation	1 – Arterial embolism requiring ampu-tation		
									20° × 2	2 – Traction			
									40° × 1	2 – Casting			
									45° × 1	1 – Patellectomy			

11	Warner 1988	Retrospective review	U.S.A	2	29	100	110° (10° to 120°)		None	1 – External fixator and casting	None	None	18 months

12	Daoud 1982	Retrospective review	Canada	6	26.5	83	85° (30° to 115°)		5° × 1	3 – Femoral fractures trea-ted with casting	None	None	12 months
										2 – Internal fixation of femoral/tibia fracture			
										1 – Osteomyelitis			

The majority of studies regarding Judet Quadricepsplasty included longitudinal cohort studies, retrospective reviews, case series and reports with the largest recent study conducted by Oliveira having a sample size of 45 patients [4]. The average follow up duration ranged from 6 months to 101 months.

The average age group for most studies ranged from 26 to 44.8 with only Bellemans [5] study reporting on outcomes of quadricepsplasty on a 64 year old for knee contracture after total knee arthroplasty. The studies were predominantly done on the male gender with an average percentage of 25% to 100% of the study population being male. The cause of contracture varied for different studies with the majority being the sequelae of traumatic periarticular injuries. Patients were treated with Ilizarov application for limb reconstruction or bone transport, external fixators, open reduction and internal fixation, prolonged traction or cast immobilisation. Unique causes of knee extension contractures were reported by Rose [6]; for severe osteoarthritis, Bellemans [5]; post total knee replacement and as a sequelae of intramuscular injections, Merchan [7]; post patellectomy for trauma and Daoud [8]; for surgically treated osteomyelitis.

The pre-operative range of motion before Judet quadricepsplasty ranged from 5° to 38°. Post quadricepsplasty range of motion ranged from 84° to 120°. The improvement in range of motion varied from 51° to 110°. Most studies reported at least one patient with extension lag post-surgery, with the degree of extension lag ranging from 5° to 45°. Bellemans [5] reported on a case of recurrence of the extension contracture eventually requiring arthrodesis. Only 3 studies[4, 7, 9] reported the need for manipulation under anaesthesia for recurrent extension contracture, with the percentage of study population requiring manipulation ranging from 14% to 70%.

Average muscular power utilizing the medical research council (MRC) grading system was 3.3 in extension and 3.9 in flexion in Mer chan’s series [7]. In his series, 6 patients retained the same amount of strength, 13 patients had increased strength and 2 had decreased strength when compared to pre-operative MRC grades. Warner’s [10] case report of a patient undergoing bilateral Judet quadricepsplasty had muscle grade of 5 on the right and 4 on the left after quadricepsplasty with excellent outcomes at 18 months. Oliveira [4] reported a slight trend towards lower strength with increasing age and lower final range of motion but did not find any correlation with patient gender.

Wound and surgical site infections were the most common complication of this procedure[4, 5, 7, 9, 11–13]. Most of the infections were superficial surgical site infections with deep infections in Belle man s and Masse’s case series originating from external fixator pin sites. One of the concerns of quadricepsplasty is the transmission of bacteria through the tissue planes as only the skin is closed, with no fascial, muscular or capsular plane closure. To mitigate the risk of infection, the authors recommend meticulous haemostasis, early mobilization and continuation of intravenous antibiotics until the drain outputs are minimal and ready for removal. None of the studies reported on the peri-procedural antibiotic regime and prophylactic treatments for prevention of infection. While closing of the tissue planes may theoretically reduce the risk of infection, this would reduce the final range of motion gained and there are no conclusive studies that demonstrate a reduction in deep infection risk with layered closure.

Masse [13] reported 1 case of quadriceps tendon rupture, 1 case of skin necrosis and 1 case of lateral femoral condyle fracture. Ali [9] had 1 complication of haematoma formation requiring surgical washout, the patient however proceeded to have a good outcome. Bellemans [5] had 1 case of compartment syndrome from constrictive dressings requiring fasciotomy. Ebraheim [14] had a case of inadequate skin coverage after removal of implants and quadricepsplasty. The patient required a primary rectus abdominis flap and immobilisation of the knee and subsequently had a poor outcome. Merchan [7] reported 1 case of arterial embolization requiring amputation 1 month after quadricesplasty.

Traumatic injuries to the knee and thigh can often result in extension contractures, especially after prolonged periods of immobilisation. Knee extension contractures may be treated conservatively initially with aggressive physical therapy and range of motion exercises. For patients who fail conservative management, surgical techniques such as manipulation under anaesthesia, arthroscopic adhesiolysis and quadricepsplasty may be considered [15]. However, in older patients with osteoporotic bones or significant contractures and fibrosis, manipulation under anaesthesia and arthroscopic adhesiolysis may be contraindicated or insufficient.

Thompson quadricepsplasty was first described in 1944 for the treatment of knee extension contractures [2]. This technique involves the detachment of the vastus medialis, vastus lateralis and vastus intermedius from the patella *via* an anterior midline approach. The rectus femoris is left intact as the sole knee extensor resulting in frequent complications of extensor lag.

The Judet technique of quadricepsplasty is broadly divided into a sequential 5 phase release of the intrinsic and extrinsic structures of the knee. The first phase involves a limited lateral incision and lateral adhesiolysis. The second phase involves a medial incision with adhesiolysis, release of the medial capsule and medial collateral ligaments. The third phase involves an extension of the lateral incision for release of the rectus femoris. The fourth phase is the quadriceps slide and the fifth phase is the fractional lengthening of the fascia lata and fascia of the vastus lateralis. This allows the surgeon to limit the procedure at any phase once adequate flexion is obtained thus preventing further soft tissue dissection and potential risk of greater extensor lag from over release. Judet initially described the release of the rectus femoris through an inguinal incision inferior to the ASIS and AIIS. Our experience shows that by extending the lateral incision, the rectus femoris can be accessed *via* the same incision, precluding the need for additional incisions.

The authors recommend a simultaneous Judet quadricepsplasty and removal of implants when there is adequate bony union as opposed to staged surgeries for soft tissue release and a delayed removal of implants for residual contracture or implant complications. While a simultaneous procedure may result in more complications such as intra-operative fractures or implant breakage, we believe this risk may be mitigated by adequate preoperative computed tomography imaging and planning. Additionally, any secondary surgery after an initial Judet quadricepsplasty may result in recurrence of scarring and contractures, undoing the good work from the previous surgery.

The extensive soft tissue and bony damage encountered in trauma patients and perhaps elements of systemic complications, chronic pain and psychological factors make management of knee contractures in trauma patients challenging. The importance of a multi-disciplinary team of orthopaedic surgeons, anaesthetists, physiotherapists, psychologists, social and familial support cannot be overstated.

Post-operative analgesia and aggressive rehabilitation are also important factors for maintaining the flexion range achieved intra-operatively. We recommend starting CPM from the postoperative care unit and the insertion of an epidural catheter for adequate pain relief. The patient should be monitored for complications such as wound site infections and precipitous drops in haemoglobin levels due to post-operative bleeding. Most authors recommend leaving the sutures for longer periods, around 3 weeks, due to the high risk of skin edge necrosis.

## 4. Conclusion

Patients with recalcitrant extension contractures of the knee may be treated surgically with the Judet quadricepsplasty. The procedure has a good track record even in older patients and can be performed in most tertiary hospitals with adequate logistical preparation and a multi-disciplinary approach.

## Compliance with ethical standards

Funding: No funding was received for the purpose of this study.

## Conflicts of interest statement

The authors declare no conflicts of interest.

## Ethical approval

All procedures performed in studies involving human participants were in accordance with the ethical standards of the institutional and/or national research committee and with the 1964 Helsinki declaration and its later amendments or comparable ethical standards. The study was approved by the institutional review board with reference number 2017/00114.

## Informed consent

Informed consent was obtained from all individual participants included in the study.
